# American Academy of Dental Science—Change of Time of Meeting

**Published:** 1878-08

**Authors:** Geo. T. Moffatt

**Affiliations:** Corresponding Secretary.


BOSTON, July 16th, 1878.

*To the Editor of the Amer. Jour. of Dental Science:*

DEAR SIR:—The time of annual meeting of the American Academy of Dental Science, has been changed from the last Monday in September, to the last Wednesday in October, believing this to be a more favorable time for securing a large attendance.

Members and others will please take notice of this change.

GEO. T. MOFFATT, *Corresponding Secretary.*

